# Initial Experience With Implantation Left Bundle Branch Pacing Leads Without a Dedicated Three‐Dimensional Sheath

**DOI:** 10.1111/pace.70039

**Published:** 2025-09-16

**Authors:** Cord‐Friedrich Niehaus, Guram Imnadze, Thomas Eitz, Heinrich Weglage, Vanessa Sciacca, Philipp Lucas, Lilit Antonyan, Sebastian E. Beyer, Ersan Akkaya, Elias Waezsada, Yuri Bocchini, Mustapha El Hamriti, Stephan Winnik, Denise Guckel, Maxim Didenko, Moneeb Khalaph, Christian Sohns, Philipp Sommer, Thomas Fink

**Affiliations:** ^1^ Clinic for Electrophysiology, Herz‐ und Diabeteszentrum NRW, Ruhr‐Universität Bochum, Bad Oeynhausen, Germany Herz‐ und Diabeteszentrum NRW Ruhr‐Universität Bochum Bad Oeynhausen Germany; ^2^ Clinic For Internal Medicine/Cardiology St. Marienhospital Vechta Vechta Germany; ^3^ Clinic for Thoracic and Cardiovascular Surgery Herz ‐und Diabeteszentrum NRW Ruhr‐Universität Bochum Bad Oeynhausen Germany; ^4^ Clinic For Internal Medicine/Cardiology and Intensive Care Medicine Niels‐Stensen‐Kliniken Marienhospital Osnabrück Osnabrück Germany; ^5^ Clinic For Cardiology and Angiology GZO Spital Wetzikon Wetzikon Zurich Switzerland

**Keywords:** implantation technique, left bundle branch pacing, stylet‐driven

## Abstract

**Aims:**

Left bundle branch area pacing (LBBAP) has emerged as an alternative to cardiac stimulation via right ventricular pacing and cardiac resynchronization therapy using coronary sinus leads. The approach utilizes dedicated three‐dimensional guiding catheters for lead placement. Our objective was to evaluate the feasibility and safety of a simplified approach of implantation of an LBBAP electrode without a dedicated guiding catheter.

**Methods:**

This was a prospective single‐center proof‐of‐concept evaluation. Patients with an indication for dual‐chamber pacemaker implantation were consecutively enrolled. All patients received ventricular lead placement with a commercially available stylet‐driven pacemaker lead. LBBAP was attempted without the use of a dedicated guiding catheter but with the help of a manually three‐dimensionally pre‐curved stylet.

**Results:**

A total of 24 patients were analyzed. Procedure and fluoroscopy durations were 61 ± 12 min and 7.4 ± 3.9 min, while LBBAP lead placement was successful in 19 patients (79%). In these patients, the V6‐R‐wave peak time was 74 ± 11 ms, the V1V6 interpeak interval was 51 ± 11 ms, and QRS width during unipolar stimulation was 123 ± 14 ms. No complications attributed to the transseptal route of the pacing lead occurred. After a mean follow‐up of 104 ± 20 days, there was no significant change in QRS widths (123 ± 15 ms, *p* = 0.94), V6‐R wave peak time (70 ± 11 ms, *p* = 0.3), and V1V6 interpeak interval (45 ± 10 ms; *p* = 0.12).

**Conclusion:**

Implantation of an LBBAP electrode without the use of a dedicated three‐dimensional sheath is feasible and safe in a high proportion of patients. Further studies are necessary to define the impact of this technique for potential use in clinical routine.

## Graphical abstract

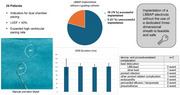

## 1 Introduction

Left bundle‐branch area pacing (LBBAP) has been shown to be an alternative to conventional dual‐chamber pacing with right ventricular (RV) lead positioning [1, 2]. Some data also suggest that LBBAP is superior to biventricular pacing by a cardiac resynchronization (CRT) system with coronary sinus (CS) leads, both in terms of heart failure therapy [3, 4] and arrhythmic events [5].

The approach utilizes dedicated three‐dimensional guiding catheters for lead placement. Procedural complexity, as well as high costs for specialized implantation tools, may prevent broader use of this technique. Our objective was to evaluate the feasibility and safety of an implantation technique of a LBBAP electrode placement without a dedicated guiding catheter, but with utilization of a manually shaped stylet.

## 2 Methods

### 2.1 Patient Population

This was a prospective single‐center proof‐of‐concept study. The study was approved by the local ethics board (ethical review board file number 2024–1219) according to the Declaration of Helsinki. Patients in whom a dual‐chamber pacemaker was indicated due to high‐grade AV block were consecutively included in the study. The predefined inclusion criteria were a dual‐chamber pacemaker indication according to the current guidelines, an LVEF > 40% and an expected high ventricular stimulation rate (>20%).

All patients received an ECG and transthoracic echocardiography preoperatively. The implantations were carried out by two different implanters with high experience in device care (>200 pacemakers and defibrillator implantations annually) and previous experience in CRT and LBBAP.

### 2.2 Implantation Procedure Workflow

**FIGURE 1:**
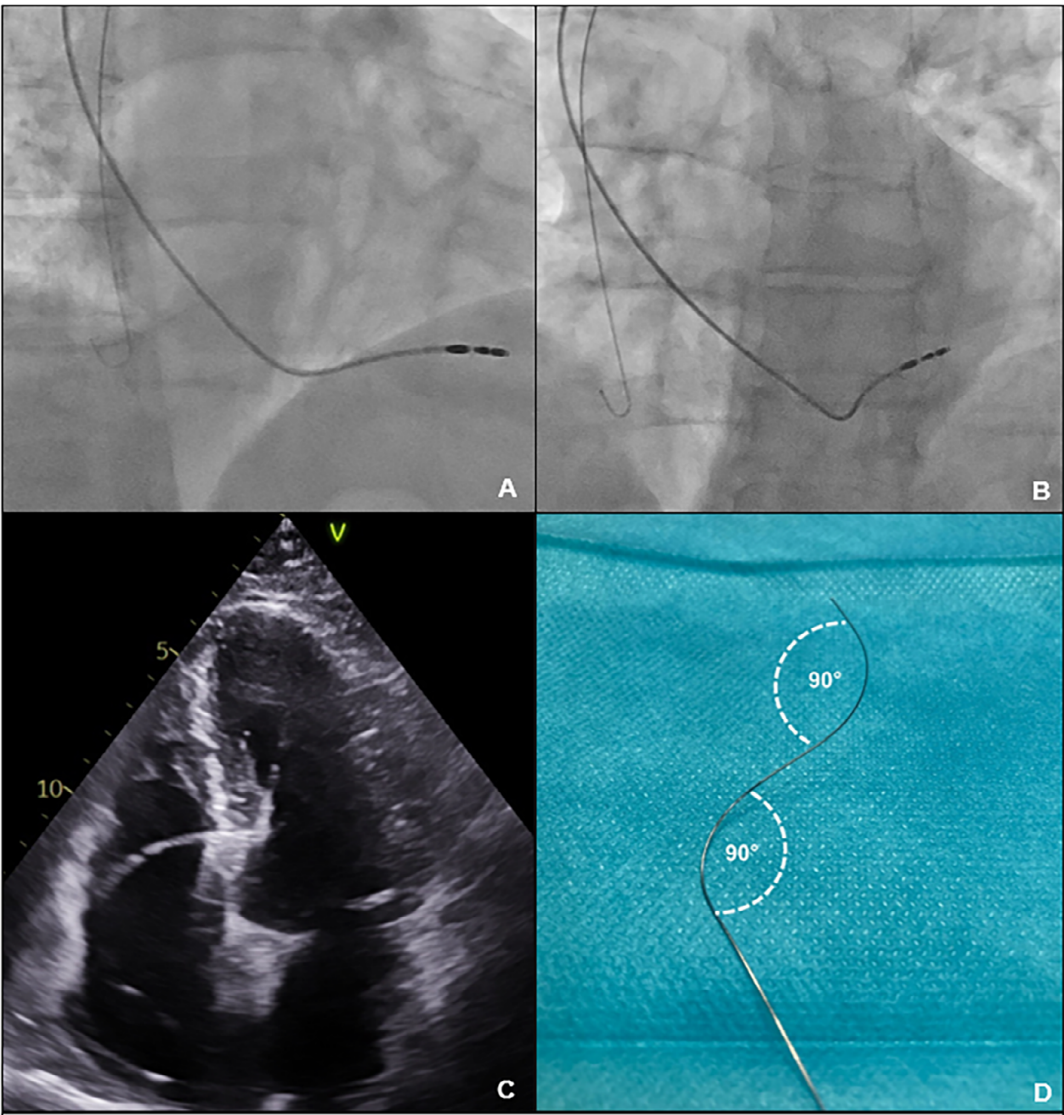
Representative images of LBBAP implantation using an implantation technique with a pre‐bent 3‐dimensional stylet. Fluoroscopy of lead positioning in RAO 20° (A) and LAO 30° (B). (C) Transthoracic echocardiography demonstrating a septal lead position. (D) A manually shaped stylet was used for lead placement. [Colour figure can be viewed at wileyonlinelibrary.com]

In all patients, a 53‐cm bipolar endocardial lead (Solia S54, Biotronik, Berlin, Germany) with active fixation was implanted in the atrium, and a commercially available 60 cm electrode (Solia S60, Biotronik) was placed in the ventricle. Solia leads are bipolar electrodes with an electrically active fixing screw and a steroid‐coated tip. The electrode connector is an IS‐1 connection. The outer diameter of the electrode is 5.9F. The electrode is guided via a stylet. In all patients, the implantation was performed without the use of a dedicated three‐dimensional catheter. In all patients, LBBAP was attempted. At the beginning of the implantation of the ventricular electrode, it was positioned in the right ventricular outflow tract or the pulmonary artery. The inserted stylet was then removed, and a pre‐curved three‐dimensional stylet was inserted (Graphical Abstract and Figure 1 A,B,D). This corresponds to the morphology of the usual guide catheters. Because the application of contrast medium into the RV via the catheter is not possible, the location of the summit of the tricuspid valve (TVA summit) must be estimated based on the movement of the inserted electrode and by detecting an annulus signal in unipolar sensing. Lead placement was performed in 20° right anterior oblique (RAO) and 30° left anterior oblique (LAO) fluoroscopy projections. As the procedure progressed, the electrode was withdrawn to approximately 15–35 mm apical to the tricuspid valve. Septal positioning was achieved by rotating the electrode counterclockwise in LAO projections during unipolar pacing via the lead stylet. At RV implantation positions, a polarity discordance in leads II and III and a W‐pattern in V1 during unipolar pacing were expected. The position of the electrode at 30° LAO is then checked. When the electrode is positioned orthogonally to the septum, the fixing screw is screwed in. The lead was advanced by rotating the cap on the distal end of the electrode five times clockwise. The electrode was then screwed into the septum by rotating it clockwise while fixing the stylet. The electrode was advanced until a right bundle branch block‐like morphology developed in V1 (Qr/QR/qR). The V6 RWPT and the V6–V1 interpeak interval are determined as described previously by Burri et al. [6].

### 2.3 Post‐Procedural Care and Clinical Follow‐Up

Periprocedural data (pacing‐threshold, sensing‐threshold, and pacing impedance) were collected. A 12‐lead ECG was also performed intraoperatively under ventricular stimulation. Postero‐anterior and lateral chest x‐rays were performed 2 h after implantation procedures and on the first post‐operative day. Pacemaker interrogation was performed before patients’ discharge. After 3 months, clinical follow‐up investigations were performed, including 12‐lead ECG during ventricular pacing and pacemaker interrogation. Transthoracic echocardiography was performed to analyze left ventricular ejection fraction (LVEF) and global longitudinal strain (GLS) via two‐dimensional speckle tracking before implantation and 3 months after.

### 2.4 Statistics

Continuous variables were expressed as mean ± standard deviation (SD) for normal distributions or as median/interquartile range (IQR) for non‐normal distributions or categorical data. Categorial variables were displayed as counts (%). Differences among groups were investigated via Student's *t*‐test. All analyses were performed using MS Excel (Microsoft Corporation, USA). Only patients with successfully implanted LBBAP lead positions were included in the statistical analysis during follow‐up investigations.

## 3 Results

### 3.1 Baseline Data and Procedural Data

A total of 24 patients underwent pacemaker implantation with LBBAP using a pre‐bent stylet between February and June 2024. Baseline characteristics are shown in Table 1. Indication for pacemaker implantation was sinus node disease in four cases (16.7 %) and AV block in 20 cases (83.3%). Four patients (16.7%) had complete left bundle branch block before implantation. The mean LVEF previous to implantation was 54.8 ± 7.7% (Table 2).

**TABLE 1:** Baseline characteristics.

Parameter	Total cohort (*n* = 24)	Successfully implanted (*n* = 19)
Male, *n* (%)	16 (66.6)	14 (73.7)
Female, *n* (%)	8 (33.3)	5 (26.3)
Age (years)	72.9 ± 15.1	75.0 ± 15.4
LV‐EF (%)	54.8 ± 7.7	55.7% ± 6.6%
LVEDD (mm)	47 ± 6	47 ± 6
LVEDV (mL)	114 ± 23	112 ± 20
LVESV (mL)	49 ± 13	40 ± 13
GLS	−16.9 ± 2.5	−17.33 ± 2.64
E/e'	14.8 ± 6.2	14.6 ± 5.6
IVSD (mm)	10.8 ± 1.8	10.9 ± 1.8
QRS‐duration (ms)	120 ± 35	120 ± 38
Left‐bundle‐branch‐block, *n* (%)	4 (16.66)	2 (10.5)
Right‐bundle‐branch‐block, *n* (%)	6 (25)	5 (26.3)
NHYH‐class	2.15 ± 0.65	2.16 ± 0.67
AV‐block III°, *n* (%)	15 (62.4)	11 (57.9)
AV‐block II°, *n* (%)	5 (20.8)	5 (26.3)
Sick‐sinus‐syndrome, *n* (%)	4 (16.6)	3 (15.8)
Brady‐tachy‐syndrome, *n* (%)	2 (8.3)	1 (5.3)
Trifscicular block (AVB I°, LAHB, RBB), *n* (%)	1 (4.2)	1 (5.3)

Abbreviations: GLS, global longitudinal strain; IVSD, enddiastolic interventricular septum diameter; LVEDD, left ventricular enddiastolic diameter; LVEDV, left ventricular enddiastolic volume; LV‐EF, left ventricular ejection fraction; LVESV, left ventricular end systolic volume; NHYH‐class, New York Heart Association classification.

**TABLE 2:** Procedural characteristics.

Total cohort (*n* = 24)	
Successful implantation	19 (79%)
Operation duration (min)	61 ± 12
Fluoroscopy duration (min)	7.4 ± 3.9
Number of positionings	1.52 ± 0.58

In the entire patient cohort, the implantation time was 61 ± 12 min (Table 2). The average fluoroscopy time was 7.4 ± 3.9 min. In a total of 20 patients, implantation was conducted via the cephalic vein (83.3%). The axillary vein was used in three (12.5%) patients and the subclavian vein in one (4.2%) patient. On average, 1.52 ± 0.58 electrode positioning attempts were made. LBBA‐pacing was successful in 19 of 24 patients (79%) (Table 2).

The duration of the QRS complex in the successfully implanted group was 120 ± 38 ms preoperatively. The intraoperative duration of the paced QRS complex in patients with successful LBBAP was 120 ± 38 ms. The average intraoperative V6 R‐wave peak time (V6 RWPT) as an expression of left ventricular excitation was 74 ± 11 ms in patients with successful LBBAP. The V1V6 interpeak interval was 51 ± 11 ms at implantation. The stimulation impedance dropped significantly from 470 ± 106 ohms on day of implantation to 390 ± 68 ohms at the 3‐month follow‐up (*p* = 0.01); however, this was not associated with a significant change in pacing‐threshold or sensing values.

### 3.2 Procedural Safety

Regarding the safety of the procedure, it was shown that the patients who were successfully treated with an LBB electrode did not experience any complications associated with this electrode (Table 3). In one patient, a device repositioning was necessary due to pain in the area of ​​the pacemaker pocket. In another patient, the right atrial electrode had to be revised due to early dislocation.

**TABLE 3:** Procedural safety.

Device‐ and procedure‐related complications	*n* = 2
Lead dislocation	
Ventricular lead	0 events
Atrial lead	1 event
Pocked infection	0 events
Other pocket‐related complications	1 event
Endocarditis	0 events
Pericardial effusion/Tamponade	0 events
Pneumothorax	0 events

### 3.3 Follow‐Up Data: Electrical Parameters

Patients successfully treated with an LBB system were included in the follow‐up. Nineteen patients received a follow‐up on the first day post‐implantation, and 18 patients (94.7%) after 3 months.

**FIGURE 2:**
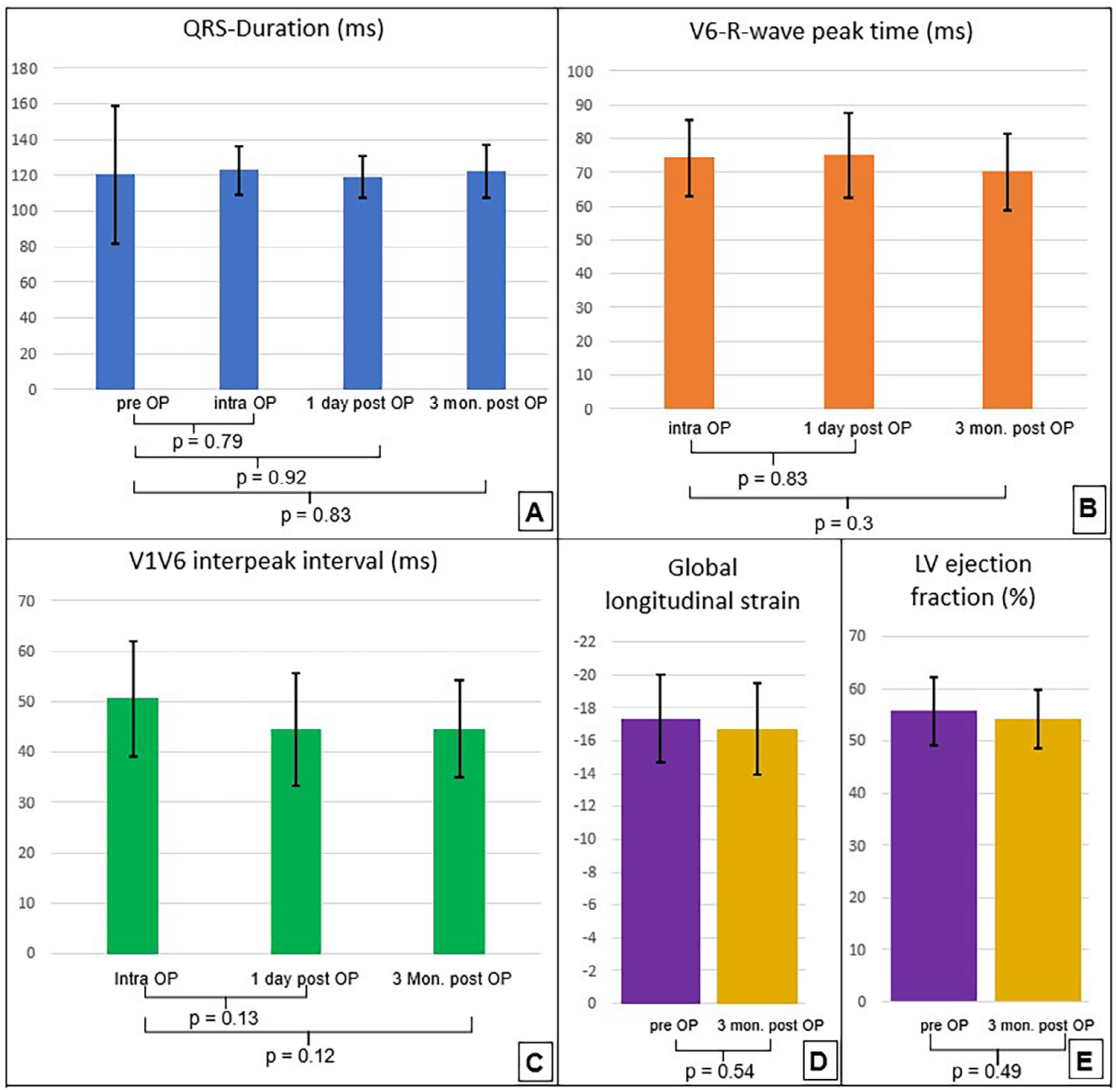
LBBAP lead parameters and echocardiographic parameters. Electrical parameters of patients with successful LBBAP lead placement (*n* = 19). (A) QRS duration preoperative, intraoperative, 1 and 3 months after implantation procedure. (B) V6‐R wave peak time preoperative, intraoperative, 1 and 3 months after implantation procedure. (C) V1‐V6 interpeak interval preoperative, intraoperative, 1 and 3 months after implantation procedure. (D) Global longitudinal strain of the left ventricle before implantation and 3 months after implantation. (E) Left ventricular ejection fraction before implantation and 3 months after implantation. [Colour figure can be viewed at wileyonlinelibrary.com]

There was no significant change of QRS width under unipolar stimulation on the day of the operation (123 ± 14 ms) or on the following day (119 ± 12 ms) or in the follow‐up after 3 months (123 ± 15 ms) (Figure 2A).

**FIGURE 3:**
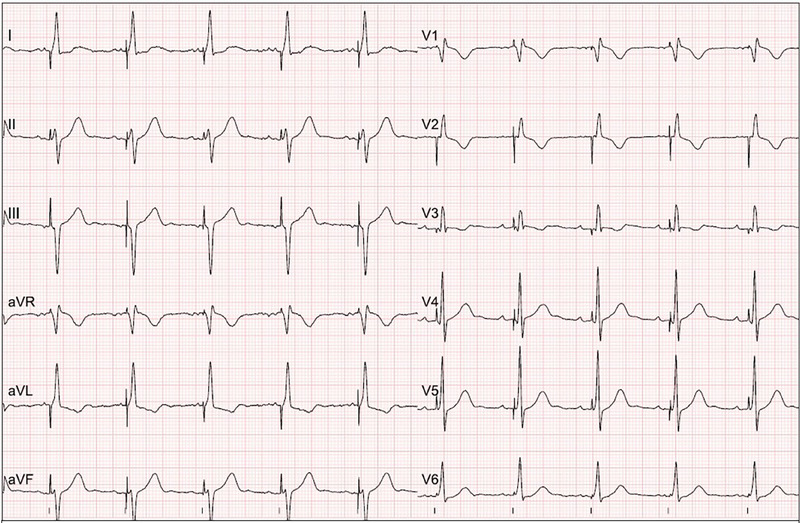
Representative ECG after successful LBBAP lead implantation. ECG during unipolar stimulation (25 mm/s). Short V6‐R wave peak time as an expression of rapid left ventricular activation and right bundle branch block morphology as an expression of delayed right ventricular activation; QRS duration was 120 ms. [Colour figure can be viewed at wileyonlinelibrary.com]

V6 RWPT remained stable after 1 day (75 ± 12 ms) and 3 months (70 ± 11 ms) (Figure 2B). The V1V6 interpeak interval showed no significant change during follow‐up after 1 day (45 ± 11 ms) and 3 months (45 ± 10 ms) (Figure 2C). The other electrical parameters were excellent on the day of implantation, as shown in Table 4, and were also stable in the follow‐up (Table 5).

**TABLE 4:** Significance analysis of perioperative values.

Parameter	Day of implantation (*n* = 19)	1. post‐operative day (*n* = 19)	3 month‐follow‐up (*n* = 18)
Loss of capture of LBB‐stimulation		0	0
Capture threshold LBB, unipolar (V)	0.54 ± 0.15	0.47 ± 0.10 *p* = 0.13	0.61 ± 0.10 *p* = 0.14
Sensing threshold LBB, bipolar (mV)	11.51 ± 4.68	12.15 ± 7.12 *p* = 0.76	13.94 ± 6.85 *p* = 0.24
Pacing impedance LBB, unipolar (Ohm)	470 ± 106	423 ± 84 *p* = 0.15	390 ± 68 *p* = 0.01
Capture threshold RA, bipolar (V)	0.8 ± 0.24	0.65 ± 0.22 *p* = 0.10	0.83 ± 0.29 *p* = 0.81
Sensing threshold RA, bipolar (mV)	2.48 ± 1.18	3.09 ± 1.21 *p* = 0.14	3.67 ± 1.43 *p* = 0.01
Pacing impedance RA, bipolar (ohm)	544 ± 58	527 ± 65 *p* = 0.40	577 ± 77 *p* = 0.16
QRS‐duration under LBB‐pacing (ms)	123 ± 14	119 ± 12 *p* = 0.42	123 ± 15 *p* = 0.94
V6‐R‐wave peak time (ms)	74 ± 11	75 ± 12 *p* = 0.83	70 ± 11 *p* = 0.30
V1V6 interpeak interval (ms)	51 ± 11	0.47 ± 0.10 *p* = 0.13	45 ± 10 *p* = 0.12

**TABLE 5:** Echocardiographic parameters.

*n* patients with follow‐up	18 (94.7%)	
Mean follow‐up (days)	104 ± 20	
NYHA‐Class	1.83 ± 0.6	*p* = 0.14
LV‐EF (%)	54.2 ± 5.6	*p* = 0.49
LVEDD (mm)	47 ± 8	*p* = 0.82
LVEDV (mm)	101 ± 23	*p* = 0.16
LVESV (mm)	44 ± 15	*p* = 0.25
GLS	−16.7 ± 2.8	*p* = 0.54
E/e'	13.5 ± 5.8	*p* = 0.63
IVSD (mm)	11.2 ± 1.7	*p* = 0.56

*Note*: Electrical parameters on the day of implantation, 1. post‐operative day, and at 3 month‐follow‐up.

Abbreviations: GLS, global longitudinal strain; IVSD, enddiastolic interventricular septum diameter; LVEDD, left ventricular enddiastolic diameter; LVEDV, left ventricular enddiastolic volume; LV‐EF, left ventricular ejection fraction; LVESV, left ventricular end systolic volume; NHYH‐class, New York Heart Association classification.

Pacing impedance dropped significantly to 390 ± 68 ohms at the 3‐month follow‐up as compared to implantation procedures (*p* = 0.01). All other parameters remained stable at the 3‐month follow‐up. The mean amount of ventricular pacing after 3 months was 76.6% ± 36%. No patient experienced a total loss of capture or loss of LBBAP stimulation during the observation period.

### 3.4 Follow‐Up Data: Echocardiography Analysis

After 3 months, no significant decrease in LVEF could be documented (55.7% ± 6.6% vs. 54.2% ± 5.6%, *p* = 0.49) (Table 5). The GLS remained stable (−17.33 ± 2.64 vs. −16.73 ± 2.81, *p* = 0.54) (Figure 2D,E). No tricuspid valve pathology was documented (Figure 3).

## 4 Discussion

The main findings of our report on LBBAP implantation using a standard pacemaker lead without a dedicated guiding catheter are that (1) the procedure is feasible with a high proportion of successful LBBAP lead implantations, (2) the procedure appears to be safe, and (3) electrical parameters and LBBAP are stable over a short‐term follow‐up of 3 months.

Mafi‐Rad et al. [7] showed for the first time in an in‐human study that the implantation of an electrode for left ventricular septal stimulation is possible via a ventricular transseptal access in 2016. In 2017, Huang et al. [8] demonstrated that stimulation of the left ventricular conduction system is possible via this access route.

The implantation of the LBBAP electrode differs fundamentally from that of a conventional lead for RV pacing, as described in a recent EHRA consensus statement by Burri et al. [6]. Current recommendations recommend the use of a dedicated three‐dimensional guiding catheter for the implantation of an LBBAP electrode. In November 2024, Ludwig et al. showed in a retrospective study the fundamental possibility of implanting an LBBAP electrode using a manually pre‐bent stylet [9]. The inclusion criteria of patients in our prospective study were previously defined. After successful LBBAP lead placement, we found favorable electrical parameters both during implantation and follow‐up, as well as stable and sustained LBBAP. The exact location of the ideal right ventricular position is very important for successful implantation. The paced QRS morphology (polarity discordance of leads II and III and V1 nadir notch) should be considered here. In the MELOS study, Jastrzebski et al. achieved a primary success rate of LBBAP implantations of 89.6% using dedicated guiding catheters in a cohort of patients with a relatively high amount of heart failure patients (27.5 %) [10]. In that study, the most frequent reason for implantation failure was inability to penetrate the interventricular septum (41.8%). In our cohort, we were successful in 79% of patients. A main reason for the difference in LBBAP success may be that there is less backup for the lead during penetration of the interventricular septum. The authors believe that this problem can be addressed by switching to a harder, more stable stylet.

In this study, we describe our initial experience with a new approach to left bundle branch area pacing (LBBAP) lead implantation utilizing a pre‐shaped stylet without the use of a dedicated delivery sheath. This technique has previously been described in both a small case series and a retrospective study.

Sun et al. investigated 20 patients who underwent LBBAP using a pre‐bent stylet from a different manufacturer and compared the results with 20 patients who underwent standard LBBAP using a lumenless lead, as well as 20 patients who received right ventricular septal pacing with an active fixation lead, following propensity score matching [11]. In that study, the success rate of the stylet‐only LBBAP approach was 92%, and electrical parameters remained stable over a 6‐month follow‐up period [11]. No significant differences were observed compared to the standard LBBAP implantation using a delivery sheath and a lumenless lead [11].

The approach using pre‐bent three‐dimensional stylets was also recently evaluated in a retrospective study by Ludwik et al. [9]. In this analysis, LBBAP outcomes were compared between patients who underwent a purely stylet‐based approach and those who underwent the standard approach, in a cohort of 230 matched individuals [9]. Ludwik et al. found comparable success rates of conduction system capture between the novel stylet‐based approach and the standard technique (95.0% vs. 94.8%), as well as similar procedure and fluoroscopy times and electrical parameters [9]. Notably, no follow‐up data were reported in this study, and the comparison involved two patient cohorts from distinct and independent implantation centers [9].

Our findings are consistent with the aforementioned studies, particularly regarding the high success rate of LBBAP implantation using a pre‐bent stylet‐only approach. Although slightly higher success rates were reported in the two cited studies, comparison across different retrospective analyses is inherently limited. It is well recognized that LBBAP implantation success is influenced by both operator experience and patient characteristics [12].

There are several potential areas where the stylet‐only approach may offer clinical value. It could serve as a cost‐effective alternative to the standard method in regions with limited access to dedicated implantation sheaths. Moreover, this approach may facilitate broader application of LBBAP, including in patients with preserved left ventricular ejection fraction (LVEF). Because pacemaker‐induced cardiomyopathies may also occur in patients with preserved left ventricular ejection fraction, a possible use of this technique may be that patients with normal LVEF and a high (>20%) expected ventricular pacing rate can receive an attempt of LBBAP in this way without the use of expensive material [13]. In our cohort, there was no significant decrease in LVEF even with a high proportion of ventricular pacing. It is known that GLS is an early indicator of the occurrence of pacemaker‐induced cardiomyopathy [14]. We found stable GLS in our patient population after 3 months. In summary, there was no evidence of the occurrence of pacemaker‐induced cardiomyopathies after a short‐period follow‐up.

Implantation procedures in our cohort were safe, effective, and potentially more cost‐effective than using a dedicated LBBAP catheter. In case of a failed implantation, a catheter can be used, if necessary.

### 4.1 Limitations

The main limitations of this study are the moderate number of patients and the short follow‐up. Further investigations should follow here. Furthermore, the study population does not include any patients with severely reduced left ventricular ejection fraction.

## 5 Conclusion

We demonstrate the feasibility, safety, and short‐term effectiveness of LBBAP without the use of a dedicated three‐dimensional sheath. Due to lower costs, this may open up the use of this technique to a wider group of patients. Further studies are important and desirable in this context.

## Ethics Statement

The study was approved by the local ethics board (ethical review board file number 2024–1219) according to the Declaration of Helsinki.

## Consent

All patients signed informed consent for the procedure and perioperative and follow‐up investigations.

## Conflicts of Interest

C.‐F.N. has received consulting fees and travel grants from Boston Scientific Medizintechnik GmbH and from Abbott GmbH outside the presented work. T.F. received advisory fees from Boston Scientific. P.S. is a member of the advisory board of Abbott, Biosense Webster, Boston Scientific, and Medtronic. C.S. received lecture fees and is a consultant for Biosense Webster, Boston Scientific, and Medtronic. All remaining authors have declared no conflicts of interest.

## Data Availability

The data underlying this study will be shared on reasonable request to the corresponding author.

## References

[1] P. S. Sharma , N. R. Patel , V. Ravi , et al., “Clinical Outcomes of Left Bundle Branch Area Pacing Compared to Right Ventricular Pacing: Results From the Geisinger‐Rush Conduction System Pacing Registry,” Heart Rhythm 19, no. 1 (2022): 3–11, 10.1016/j.hrthm.2021.08.033. 34481985

[2] X. Li , J. Zhang , C. Qiu , et al., “Clinical Outcomes in Patients With Left Bundle Branch Area Pacing vs. Right Ventricular Pacing for Atrioventricular Block,” Frontiers in Cardiovascular Medicine 8 (2021): 685253, 10.3389/fcvm.2021.685253. 34307499 PMC8297826

[3] P. Vijayaraman , S. Ponnusamy , Ó. Cano , et al., “Left Bundle Branch Area Pacing for Cardiac Resynchronization Therapy,” JACC: Clinical Electrophysiology 7, no. 2 (2021): 135–147, 10.1016/j.jacep.2020.08.015. 33602393

[4] P. Vijayaraman , P. S. Sharma , Ó. Cano , et al., “Comparison of Left Bundle Branch Area Pacing and Biventricular Pacing in Candidates for Resynchronization Therapy,” Journal of the American College of Cardiology 82, no. 3 (2023): 228–241, 10.1016/j.jacc.2023.05.006. 37220862

[5] B. Herweg , P. S. Sharma , Ó. Cano , et al., “Arrhythmic Risk in Biventricular Pacing Compared With Left Bundle Branch Area Pacing: Results from the I‐CLAS Study,” Circulation 149, no. 5 (2024): 379–390, 10.1161/CIRCULATIONAHA.123.067465. 37950738

[6] H. Burri , M. Jastrzebski , Ó. Cano , et al., “EHRA Clinical Consensus Statement on Conduction System Pacing Implantation: Endorsed by the Asia Pacific Heart Rhythm Society (APHRS), Canadian Heart Rhythm Society (CHRS), and Latin American Heart Rhythm Society (LAHRS),” Europace 25, no. 4 (2023): 1208–1236, 10.1093/europace/euad043. 37061848 PMC10105878

[7] M. Mafi‐Rad , J. G. L. M. Luermans , Y. Blaauw , et al., “Feasibility and Acute Hemodynamic Effect of Left Ventricular Septal Pacing by Transvenous Approach Through the Interventricular Septum,” Circulation: Arrhythmia and Electrophysiology 9, no. 3 (2016): e003344, 10.1161/CIRCEP.115.003344. 26888445

[8] W. Huang , L. Su , S. Wu , et al., “A Novel Pacing Strategy with Low and Stable Output: Pacing the Left Bundle Branch Immediately Beyond the Conduction Block,” Canadian Journal of Cardiology 33, no. 12 (2017): 1736.e1–1736.e3, 10.1016/j.cjca.2017.09.013. 29173611

[9] B. Ludwik , M. Labus , T. Roleder , et al., “Novel Approach to Left Bundle Branch Area Pacing Lead Implantation Using a 3‐Dimensional Stylet,” Heart Rhythm S1547‐5271, no. 24 (2024): 03569–0, 10.1016/j.hrthm.2024.11.016. 39551119

[10] M. Jastrzębski , G. Kiełbasa , and O. Canoet , “Left Bundle Branch Area Pacing Outcomes: The Multicentre European MELOS Study,” European Heart Journal 43, no. 40 (2022): 4161–4173, 10.1093/eurheartj/ehac445. 35979843 PMC9584750

[11] Y. Sun , X. Yao , X. Zhou , et al., “Preliminary Experience of Permanent Left Bundle Branch Area Pacing Using Stylet‐Directed Pacing Lead Without Delivery Sheath,” Pacing and Clinical Electrophysiology 45, no. 8 (2022): 993–1003, 10.1111/pace.14504. Epub 2022 May 19.. 35437783

[12] J. Zhang , Y. Zhang , Y. Sun , et al., “Success Rates, Challenges and Troubleshooting of Left Bundle Branch Area Pacing as a Cardiac Resynchronization Therapy for Treating Patients With Heart Failure,” Frontiers in Cardiovascular Medicine 9 (2023): 1062372, 10.3389/fcvm.2022.1062372.; PMCID: PMC9872722. 36704478 PMC9872722

[13] E. L. Kiehl , T. Makki , R. Kumar , et al., “Incidence and Predictors of Right Ventricular Pacing‐Induced Cardiomyopathy in Patients With Complete Atrioventricular Block and Preserved Left Ventricular Systolic Function,” Heart Rhythm 13, no. 12 (2016): 2272–2278, 10.1016/j.hrthm.2016.09.027. 27855853

[14] K. Manocha , M. S. Kandola , R. Kalil , et al., “Reduction of Left Ventricular Global Longitudinal Strain in Patients With Permanent Pacemakers as a Predictor of Heart Failure and Mortality Outcomes,” Pacing and Clinical Electrophysiology 46, no. 5 (2023): 385–391, 10.1111/pace.14701. 37087556 PMC10288370

